# Natural Coinfection between Novel Species of Baculoviruses in *Spodoptera ornithogalli* Larvae

**DOI:** 10.3390/v13122520

**Published:** 2021-12-15

**Authors:** Gloria Patricia Barrera, Laura Fernanda Villamizar, Gustavo Adolfo Araque, Juliana Andrea Gómez, Elsa Judith Guevara, Carolina Susana Cerrudo, Mariano Nicolás Belaich

**Affiliations:** 1 Corporación Colombiana de Investigación Agropecuaria-Agrosavia, Centro de Investigación Tibaitatá, Kilómetro 14 vía Mosquera, Bogotá 250047, Cundinamarca, Colombia; garaque@agrosavia.co (G.A.A.); jagomez@agrosavia.co (J.A.G.); eguevara@agrosavia.co (E.J.G.); 2 Microbial Solutions, AgResearch Ltd., Lincoln Research Centre, Private Bag 4749, Christchurch 8140, New Zealand; laura.villamizar@agresearch.co.nz; 3 Laboratorio de Ingeniería Genética y Biología Celular y Molecular—Área Virosis de Insectos (LIGBCM—AVI), Dto. de Ciencia y Tecnología, Universidad Nacional de Quilmes, Roque Saenz Peña 352, Bernal 1876, Provincia de Buenos Aires, Argentina; ccerrudo@unq.edu.ar (C.S.C.); mbelaich@unq.edu.ar (M.N.B.)

**Keywords:** *Spodoptera ornithogalli*, *Spodoptera frugiperda*, SporNPV, SporGV, natural coinfection

## Abstract

*Spodoptera ornithogalli* (Guenée) (Lepidoptera: Noctuidae) is an important pest in different crops of economic relevance in America. For its control, strategies that include chemicals are usually used; so, the description of entomopathogens would be very useful for the formulation of biopesticides. In this regard, two different baculoviruses affecting *S. ornithogalli* were isolated in Colombia, with one of them being an NPV and the other a GV. Ultrastructural, molecular, and biological characterization showed that both isolates possess the 38 core genes and are novel species in *Baculoviridae*, named as *Spodoptera ornithogalli nucleopolyhedrovirus* (SporNPV) and *Spodoptera ornithogalli granulovirus* (SporGV). The bioassays carried out in larvae of *S. ornithogalli* and *S. frugiperda* showed infectivity in both hosts but being higher in the first. In addition, it was observed that SporGV potentiates the insecticidal action of SporNPV (maximum value in ratio 2.5:97.5). Both viruses are individually infective but coexist in nature, producing mixed infections with a synergistic effect that improves the performance of the NPV and enables the transmission of the GV, which presents a slowly killing phenotype.

## 1. Introduction

The yellow striped armyworm *Spodoptera ornithogalli* (Guenée) (Lepidoptera: Noctuidae) is a highly polyphagous insect on a wide range of botanical families that can include annual crops, vegetables, weeds, ornamental, flowers, and fruits [1,2]. It is found in the U.S.A., Mexico, Central America, and the Caribbean [3], causing important damages in crops (even with a severity of 100%) due to its elevated reproductive potential. The larvae consume leaves and fruits, producing a severe defoliation [4].

Several natural enemies for *S. ornithogalli* control have been reported, but the chemical treatment predominates as the alternative for its management, as occurs with many agricultural pests. The potential damage to ecosystems and human health that this entails encourages the development of biological control tools that can collaborate in pest integrated management. In this sense, one of the most studied control agents for lepidoptera are baculoviruses. These entomopathogenic viruses are classified according to the occlusion body (OB) morphology in granuloviruses (GVs), which usually contain a single enveloped virion in a granular OB, and the nucleopolyhedroviruses (NPVs), which can contain few to many enveloped virions in a polyhedral OB [5]. Currently, the *Baculoviridae* family is divided into four genera: *Alpha-* and *Betabaculovirus* (lepidopteran specific NPVs and GVs, respectively), *Deltabaculovirus* (NPVs in Diptera), and *Gammabaculovirus* (NPVs in hymenoptera) [6].

In 1967, an NPV was isolated in California (USA) for the first time from *S. ornithogalli* larvae. Later, similar viruses were isolated and partially characterized in other geographic regions [7,8], determining their potential for control but without reporting genomic sequence information. In Colombia, a nucleopolyhedrovirus of S. ornithogalli was collected from citric crop and was characterized determining its biological performance [9]. However, further studies demonstrated the presence of a mixed infection with a granulovirus (SporGV, previously not reported) and a nucleopolyhedrovirus (SporNPV), arising the need to understand the potential interactions of both entomopathogens. Moreover, there were no genomic data available to understand whether these isolates were genotypes of described species or were part of different species not recognized so far. 

Mixed baculovirus infections in a single insect larva are subject to study from a practical point of view when these entities are applied as biocontrol agents [10], but also to better understand the cases of natural coinfections. It is important to know if the two viruses can act independently of each other and if their effects when acting together are additive, synergistic, or antagonistic [11]. Documented viral interactions have a significant effect on the severity of viral diseases, host range, transmission, and the immunopathology, allowing us to improve the development of biological control tools, increasing the range of action and/or the insecticidal capacity of the viruses used [12]. For example, a natural coinfection in a similar insect pest, such as *S. frugiperda*, demonstrated the enhancer activity of a GV over the insecticidal activity of a NPV, increasing the pathogenicity but only using a low GV:NPV ratio [13]. From the knowledge of the interactions between entomopathogenic viruses, some ecological bases can be elucidated and the natural co-infection phenomenon can be exploited for integrated pest management programs in agriculture.

Regarding this context, the aim of the present work was to characterize the Colombian isolates Spodoptera ornithogalli granulovirus (SporGV) and Spodoptera ornithogalli nucleopolyhedrovirus (SporNPV), baculoviruses naturally occurring in coinfection processes, classify them within *Baculoviridae*, and analyze their biological effects on *S. ornithogalli* larvae to initiate the path to the development of a baculovirus-based biopesticide for *S. ornithogalli*.

## 2. Materials and Methods

### 2.1. Insect Rearing 

Larvae of *S. ornithogalli* were obtained from a laboratory colony established from insects collected in a citric crop located at Puerto López (Colombia), while larvae of *S. frugiperda* were obtained from a laboratory colony established from eggs collected in a maize crop located at Tolima (Colombia). Both colonies were refreshed every 6 months by introducing insects collected in the field. Larvae were maintained at 25 °C and 60% relative humidity (RH), under a natural photoperiod of 12:12 h (light:dark) and were fed with a wheat germ-based semi-synthetic diet [13].

### 2.2. Virus Production, Purification, and Quantification

*S. ornithogalli* larvae with viral symptoms collected in a citric crop located at Puerto López (Colombia) [9] were macerated and the obtained suspension was used to inoculate third instar (L3) larvae by the droplet feeding method [14]. Cadavers with viral signs were collected and homogenized in 0.1% (*m/V*) sodium dodecyl sulphate (SDS) solution, and the obtained suspension containing a natural viral mixture (SporNPV and SporGV) was filtered through three layers of cheesecloth [15]. Then, both baculoviruses were purified by differential centrifugation on continuous 1.17- to 1.30-g/mL sucrose gradients at 104,000× *g* for 40 min at 4 °C, and the recovered bands were used to infect second instar larvae (L2) of *S. ornithogalli* using the droplet feeding method [14]. Dead larvae with GV or NPV infection signs were collected. Each virus was purified using sucrose gradients and the collected bands were again used to infect larvae (L2), a procedure that was repeated four times. In each round of infection, only larvae that showed signs of infection were selected. The purity of each viral type was verified by transmission electron microscopy (TEM). For this purpose, starved larvae were orally inoculated with suspensions containing 106 OBs/mL. Then, larvae were maintained at 25 °C and 60% RH, under a natural photoperiod of 12:12 h (light:dark) until death. OBs were extracted from dead diseased larvae by homogenizing cadavers in 0.1% (*m/V*) SDS solution and purified by filtration and differential centrifugations. GVs’ suspensions were quantified by measuring the absorbance at 280 nm and extrapolating the concentration in a previously standardized calibration curve [16]. NPVs’ suspensions were quantified using a Neubauer hemocytometer (Hawksley Ltd., Lancing, UK) under a light microscopy at 40×.

### 2.3. Electron Microscopy

Purified OBs were fixed using 2.5% (by volume) glutaraldehyde in Phosphate-buffered saline (PBS) for 24 h at 4 °C, washed twice with PBS, and then post-fixed in 1% (*m/V*) osmium tetroxide (Electron Microscopy Sciences, Hatfield, PA, USA) for 1 h at 4 °C. After washing twice with PBS, the samples were dehydrated [ethanol 70% (by volume)], infiltrated and embedded in LR White resin (Merck, Darmstadt, Germany). Samples were sectioned and mounted onto 100-mesh copper grids (Agar Scientific Ltd., Essex, UK) and double-stained with uranyl acetate and lead citrate. Ultrathin sections were observed under a JEOL JEM 1230 transmission electron microscope (JEOL Ltd., Tokyo, Japan) at 80 kV.

### 2.4. Genome Sequencing and Analysis 

Viral DNA of SporNPV and SporGV was obtained from purified NPVs and GVs, respectively. OBs were dissolved by alkaline lysis and DNA was extracted by standard methods [15]. Whole genome sequencing was then conducted using Illumina platform (NovaSeq 6000 PE150; Novogene, Beijing, China). For quality control and trimming analysis, the FastQC and Trimmomatic programs were used [17], while Spades v. 3.13.0 in the Galaxy platform (http://usegalaxy.eu (accessed on 25 October 2021) was employed for the sequence assembly with default parameters [18]. Open reading frames (ORFs) were identified using ARTEMIS [19], the Fickett’s method [20], and BlastP [21]. The concatenated 38 core proteins from 71 baculoviruses plus the two isolates of *S. ornithogalli* (Table S1) were then used for a phylogeny inference applying the Maximum Likelihood method and the analysis “ModelFinder + tree reconstruction + ultrafast bootstrap (1000 replicates)” in IQ-TREE 2.1.2 [22]. Moreover, the evolutionary divergence analysis was performed in the MEGAX [23] using the Kimura 2-parameters (K2P) model and the 38 concatenated core genes from 20 betabaculoviruses and 26 Group II-alphabaculoviruses (Table S1) for SporGV and SporNPV, respectively [24]. To find putative functional domains in unique genes, the HHpred server was used [25].

### 2.5. Biological Activity of Natural Mixed Viruses

Neonate larvae of *S. ornithogalli* and *S. frugiperda* starved for 16 h were fed with aqueous suspensions containing 10% (*m/V*) sucrose, 0.001% (*m/V*) blue food dye Tuska^®^ (blue No. 1 and blue No. 2), and the natural viral mixture at different concentrations. The virus concentration was adjusted based on the enumeration of SporNPV OBs using the Neubauer hemocytometer (Hawksley Ltd., Lancing, UK) under a light microscopy at 40×. Viral treatments corresponded to the following SporNPV concentrations: 1.0 × 10^4^, 1.0 × 10^5^, 1.0 × 10^6^, 1.0 × 10^7^, and 1.0 × 10^8^ OBs/mL. The absolute control corresponded to insects without treatment. Larvae that ingested the suspension were transferred to individual 14-mL cups containing a piece of semisynthetic diet. The experimental unit consisted of 15 cups contained in a plastic box (21 × 20 × 5 cm). The experimental design was completely random, with three replicates (15 larvae for each one) per treatment (45 larvae). Larvae were reared at 25 °C and 60% RH. Mortality was recorded 7 days after inoculation. Virus-induced mortality was subjected to logit regression using the Generalized Linear Interactive Modeling (GLIM) program.

### 2.6. Biological Activity of Purified Viruses and Artificial Mixtures

The LC_50_ of three mixtures prepared using different ratio of purified viruses [90% SporNPV: 10% SporGV (M1); 95% SporNPV: 5% SporGV (M2); and 97.5% SporNPV: 2.5% SporGV (M3)] was determined. Each sample was adjusted to five total concentrations of viral OB (CTot): 1 × 10^4^, 1 × 10^5^, 1 × 10^6^, 1 × 10^7^, and 1 × 10^8^ OBs/mL. Additionally, suspensions individually containing SporNPV or SporGV were prepared and adjusted to the same five concentrations. All final suspensions contained 4% (*m/V*) sucrose and 1% (*m/V*) blue food dye Tuska^®^ (blue No. 1 and blue No. 2). Pathogenicity and virulence were determined on *S. ornithogalli* larvae by using the droplet feeding method [14]. The absolute control corresponded to larvae without treatment and the treated control corresponded to larvae fed only with sucrose and dye solution without containing viruses. Three groups of 10 neonate larvae (three replicates) starved for 12 h were inoculated with each viral suspension and individually transferred into 14-mL cups provided with artificial diet and reared at 25 °C and 60% RH, under a natural photoperiod of 12:12 h (light:dark). Mortality was recorded 7 days after inoculation for treatments using SporNPV and after 27 days for when it was used on SporGV alone. The experimental design was completely random, with three replicates per treatments. Mortality data of the treatments were corrected with the mortality obtained with the control treatment. The LC_50_ of each viral mixture was determined by using the logit regression using the Generalized Linear Interactive Modeling program (GLIM) [26]. The slopes were calculated by using PoloPlus program [27]. For the comparison of LC_50_ values, parallelism of the calculated lines was checked by using the parallel-line assay option. Relative potency (RP) for each mixture was determined according with SporNPV and calculated using the following formula:

RP = LC_50_ SporNPV/LC_50_ mixture.

An additive, synergistic, or antagonistic effect between SporNPV (97.5%) and SporGV (2.5%) was determined using the mortality data obtained 7 days after inoculation and the Koppenhofer and Kaya [11] formula as follow:

EM = M_SporNPV_ + M_SporGV_ (1 − M_SporNPV_)

where M corresponds to the mortalities caused by each virus alone while EM corresponds to expected mortality of the combination of both viruses. The chi-square value [χ^2^ = (MC-EM)^2^/EM] calculated using the mortality of the mixture (MC) was compared to the chi-square table value for 1 degree of freedom (χ^2^ = 3.841) (α = 0.05). Values higher than 3.841 were considered as possible synergistic (positive value) or antagonistic (negative value) effect depending on the difference value (D) between MC and EM.

## 3. Results

### 3.1. SporNPV and SporGV

#### 3.1.1. Ultrastructural Studies and Infection Signs

**Figure 1:**
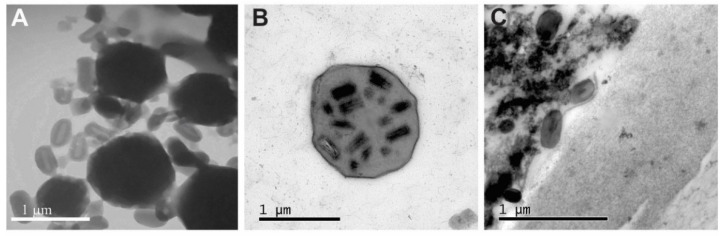
SporNPV and SporGV ultrastructural studies. Transmission electron micrographs showing OBs found in a naturally coinfected *S. ornithogalli* larva. (**A**). Natural mix of *S. ornithogalli* NPV and GV (negative staining). (**B**). *S. ornithogalli* NPV. (**C**). *S. ornithogalli* GV.

Two different baculovirus OBs were found in a naturally coinfected larva of S. ornithogalli captured in the Colombian field [9]. One of them had the typical characteristics of alphabaculoviruses (size range between 1.0–1.5 µm), while the other shared structural similarity with betabaculoviruses (range 0.25–0.40 µm in length and 0.14–0.21 µm in width), in agreement with that observed by means of electron microscopy (Figure 1). Regarding this and the insect species where both viruses were detected, they were named SporNPV and SporGV, respectively.

So far, the NPVs described in *Spodoptera* spp. from the New and the Old World belong to the Group 2-*Alphabaculovirus* genus (SeMNPV, SfMNPV, SpltNPV-II, SpliNPV and SpltMNPV-G2). SporMNPV showed the characteristics expected for these types of NPVs, disposing several nucleocapsids per ODV and showing polyhedra of conserved size [28]. Meanwhile, only two GV species have been reported for *Spodoptera* spp. worldwide (SpfrGV and SpliGV). Like the rest of betabaculoviruses described, SporGV shares dimensions and the arrangement of 1 nucleocapsid per OB.

**Figure 2:**
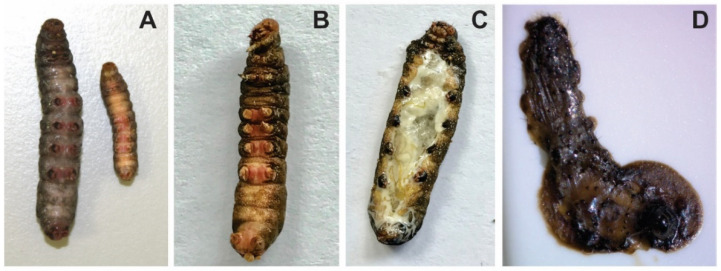
S. ornithogalli infected with SporGV and SporNPV. *S. ornithogalli* larvae have been individually infected with SporGV or SporNPV and the cadavers were photographed. (**A**). A healthy larva (on the right) next to a larva infected with SporGV (on the left). (**B**). Dead larva by SporGV. (**C**). Dead larva by SporGV with the abdomen opened with a scalpel to show its interior. (**D**). Dead larva by SporNPV.

Once both viruses could be separated in the laboratory through a careful procedure that included ultracentrifugations and successive rounds of infection, they could multiply individually in *S. ornithogalli* larvae. The signs of SporNPV infection were the traditional ones for this type of virus, showing liquefaction and death between 5 and 7 days. Meanwhile, the infection with SporGV caused death at 21 days, without external damage but showing the consequences of the infection inside (Figure 2).

Interestingly, larvae infected with the natural mixture of viruses showed the same signs as when infected with SporNPV alone. Additionally, insects also died after 5 to 7 days, suggesting that NPV drives the infection process. In view of these results, SporGV would be a typical slowly killing GV as the other ones that infect Noctuidae family [29].

#### 3.1.2. Molecular Characterization

**Figure 3:**
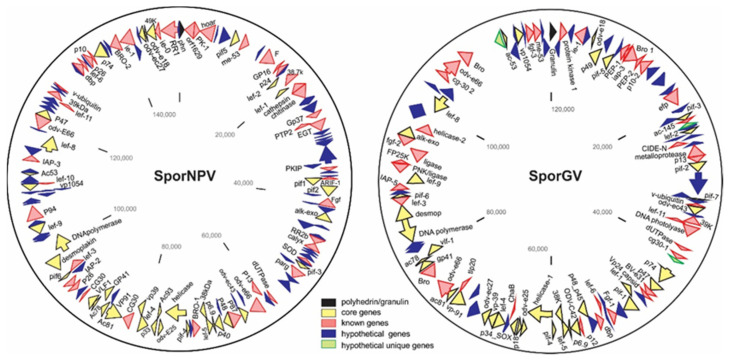
SporNPV and SporGV genomes. Physical maps of the genomes of SporNPV (**left**) and SporGV (**right**). The coding regions of genes are indicated by arrows of different colors (references are indicated).

To expand the biological information on these isolates, a molecular analysis was preformed to identify them as genotypes of reported baculoviruses or undescribed species. Viral DNA was thus recovered from virions previously isolated and amplified in *S. ornithogalli* larvae for whole genome sequencing (Figure 3).

As previously demonstrated, both viruses can individually infect S. ornithogalli larvae, which is reflected in each one by the presence of the 38 core genes [30,31] of the Baculoviridae family. SporNPV (143,756 bp; 147 ORFs) has the typical gene content of Group 2-alphabaculoviruses, showing the presence of chitinase and cathepsin genes that encode two of the enzymes responsible for the larva liquefaction, a sign that was observed in advanced stages of the infection (Figure 2D). Meanwhile, SporGV (120,202 bp; 119 ORFs) does not have these genes (correlating with the absence of larva liquefaction) and carries what is found in betabaculoviruses also having three hypothetical unique genes without similarity with other reported baculovirus sequences (ORF025, ORF047 and ORF113). Among these, ORF025 (17,390.48 Da) and ORF113 (39,199.27 Da) could translate into polypeptides with UV radiation resistance-associated and oxidoreductase motives, respectively. 

**Figure 4:**
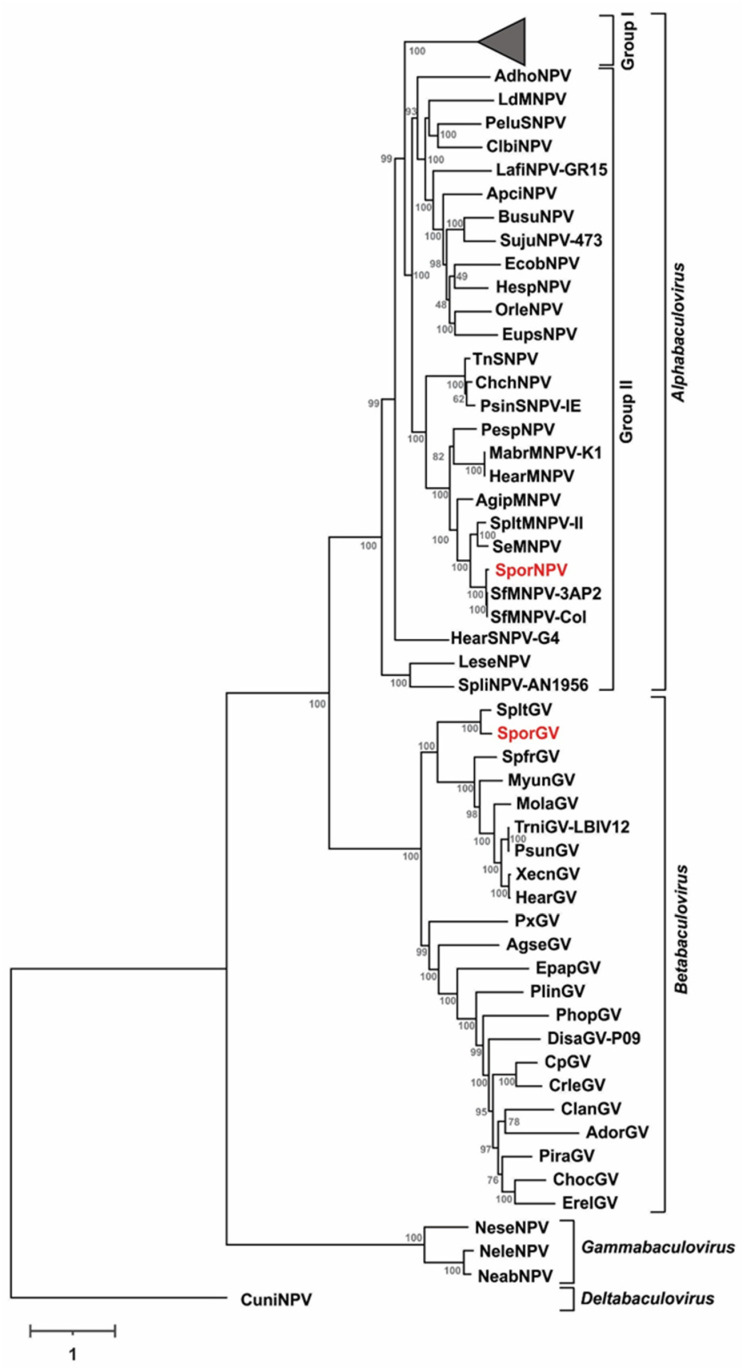
Phylogenetical inference for SporNPV and SporGV genomes. Cladogram based on a concatemer built with the 38 baculovirus core proteins. Group I-alphabaculovirus genus was collapsed to preserve space. SporNPV and SporGV are highlighted in red letters.

The phylogenetical inference confirmed that SporNPV belongs to Group 2-Alphabaculovirus genus and SporGV to Betabaculovirus (Figure 4). The tree topology was according with a previously reported analysis [24].

Like the phylogenetically closest betabaculovirus species, SpltGV, the SporGV genome did not evidence the presence of homologs to previously reported enhancing genes, enzymes involved in peritrophic membrane degradation [32]. However, among the auxiliar genes is that of the matrix-metalloprotease (MMP), also present in the rest of the betabaculoviruses but absent in the other baculovirus genera. The product of this coding sequence is a zinc-dependent endopeptidase that usually degrades extracellular matrix proteins and has been reported as a potentiating agent for the infection of NPVs [33]. Additionally, like SpltGV, SporGV contains a gene that expresses a DNA photolyase, an enzyme associated with the repair of UV damage on DNA [32]. These two GVs would be the only ones reported to carry these types of auxiliary genes, which can collaborate in natural mixtures with SporNPV in giving greater stability against solar radiation.

To investigate whether it would be appropriate to consider them as novel species, a Kimura-2P study was carried out with the 38 core genes [24] (Table 1 and Table 2).

That study reported that distances’ values higher than 0.072 between two isolates correspond to different species of baculoviruses. Regarding this, SporGV corresponds to a novel betabaculovirus species because the closest related virus (SpltGV) presented a K2P distance value of 0.309. SporNPV is related to SfMNPV showing distances between 0.021 and 0.025. According to Wennmann et al. [24], these distances are in the lower limit that serves for the demarcation of the species, although additional biological information is required for its confirmation. In this sense, it is important to highlight the morphological differences that occur between the OBs of SporNPV (1–1.5 µm) and SfMNPV (1.5–2.0 µm) [9]. Moreover, the infectivity of these viruses in their main hosts is significantly different, as shown below in the present work. Furthermore, attempts made to expose *S. ornithogalli* larvae with different concentrations of SfMNPV showed no signs of an infection progress (data not shown). This body of evidence supports the consideration of SporNPV as a novel species of Group II Alphabaculovirus. 

### 3.2. SporNPV and SporGV Biological Activity

#### Insecticidal Activity of Wild Viral Mixture

Insecticidal activity of the wild viral mixture after one amplification on *S. ornithogalli* larvae was determined in a bioassay against its original host *S. ornithogalli* and against *S. frugiperda*. Mortality in *S. ornithogalli* reached 100% with the highest doses of 1.0 × 10^7^ and 1.0 × 10^8^ OBs/mL, while maximum mortality in *S. frugiperda* was only 48% with the highest dose (1.0 × 10^8^ OBs/mL). All dead larvae presented typical symptoms of polyhedrosis disease (Figure 2D), characterized by a brownish color, waxy appearance, tegument fragility, and cadaver liquefaction. The LC_50_ of the wild natural mixture in neonate larvae of *S. ornithogalli* is presented in Table 3, a parameter that could not be estimated for *S. frugiperda* because mortality did not exceed 50%. 

Moreover, to determine the effect of SporGV on the biological activity of SporMNPV against neonate larvae of *S. ornithogalli*, a dose-response bioassay with three artificial mixtures of both viruses previously isolated was conducted, in addition to studying the effects that each entomopathogen separately produces in insects. Thus, larvae infected only with SporGV slowly developed the typical granulosis disease symptoms (decrease in mobility, whiteish coloration, and swollen body due to the accumulation of OBs), as previously was shown (Figure 2B,C), and survived for at least 3 weeks, with non-ruptured integument after death. Maximum mortality after 7 days of inoculation was obtained with the highest dose (1.0 × 10^8^ OBs/mL) with 6.66%, a value that continued increasing until 46.6% after 3 weeks. Due to the low mortality recorded at day 7, the LC_50_ could not be estimated.

By contrast, larvae infected only with SporMNPV or the viral mixtures developed symptoms of nucleopolyhedrosis disease and died in less than 1 week. Generated logit regressions showed a direct relationship between dose and mortality. Parallelism of regression lines for the mixtures and the SporMNPV was determined to calculate the relative potency (RP). Mean lethal concentrations and relative potencies are presented in Table 4.

The mixture M3 containing 97.5% of SporNPV and 2.5% of SporGV showed the highest insecticidal activity, being the only treatment with a LC_50_ significantly lower (*p* < 0.01) than that obtained for SporNPV alone. This result was related to a synergistic effect between both viruses SporNPV and SporGV (97.5:2.5 ratio; χ^2^ = 12.06, df = 1; D = 28%).

**Figure 5:**
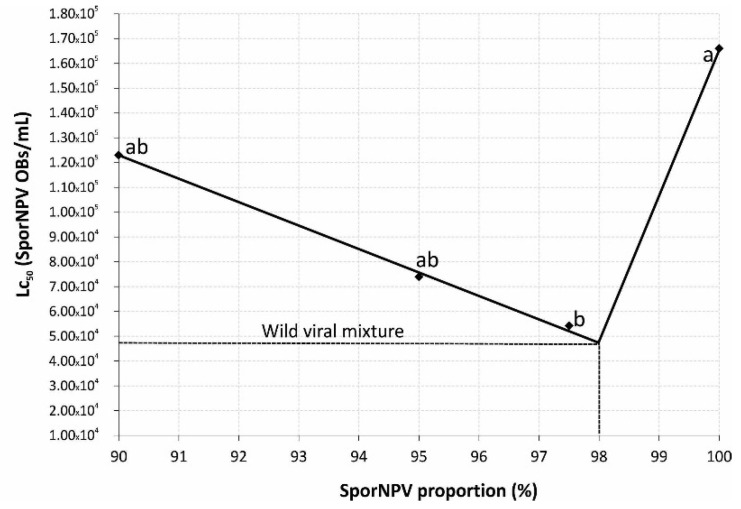
Mean lethal concentrations of artificial mixtures of SporNPV and SporGV. LC50 values followed by different letters are significantly different according to fiducial limits’ comparison. The dotted line corresponds to the extrapolation of SporNPV proportion in the wild viral mixture based in its LC_50_. Dots with the same letter did not present significant differences based on fiducial limit comparisons.

On the other hand, the proportion of SporNPV in the wild viral mixture was extrapolated in a curve relating LC_50_ values vs. the SporNPV proportion used in the artificial mixtures (Figure 5), obtaining that the natural mixture found in the field contained approximately 98.0% of SporNPV and 2.0% of SporGV.

## 4. Discussion

The wild viral mixture of SporNPV and SporGV was pathogenic to *S. frugiperda* larvae, but it was more virulent against its original host *S. ornithogalli*. Baculoviruses normally have a narrow host range, the reason why they are generally regarded as exceedingly safe biological control agents [34]. However, biocontrol agents with wider host ranges are preferred due to practical and commercial advantages. There is a trade-off between environmental concerns, where a very narrow host range is preferred, and practical and commercial interests, which require that a product deals with all pest species found on a specific crop [35]. The insecticidal activity obtained in the present work against the alternative host *S. frugiperda* suggests that SporNPV and SporGV can infect other species, being necessary to assess its pathogenicity against other pests and non-target insects. 

The artificial mixture containing 97.5% of SporNPV and 2.5% of SporGV presented the highest relative potency, being three times more potent than SporNPV used individually. The same ratio of viruses was found by Cuartas et al. [13] as the most potent mixture of SfMNPV and SpfrGV against *S. frugiperda*; they also found that virulence reduced when the proportion of GV increased. Obtained result confirmed that the interaction between NPVs and GVs leads to an increase in efficacy, an effect that has been previously demonstrated for several viral species as the NPV and GV of *S. frugiperda* [13], *Spodoptera litura* [36], and *Spodopera exigua* [37]. Virus–virus interactions in co-infected hosts may involve: (1) direct interactions among genes or gene products of the viruses; (2) indirect interactions through alteration of the host environment; and (3) indirect interaction by modulation of both innate and induced host immune responses [38]. However, the enhancing effect of GVs over NPVs has been mainly related with several proteins contained in the OBs of different granuloviruses, which can digest and/or disturb the complex of proteins and chitin that composes the peritrophic membrane from infected insects by altering its structural integrity and increasing its permeability. This allows a faster entry of the viral particles into the cells, which speed up the infection process [13,39,40,41]. This is an indirect viral interaction classified in the subtype described by Dapalma [38] as “altered host susceptibility due to breakdown of physical barriers”. Although the SporGV genome does not show homologs to the typical enhancins of betabaculoviruses, it does possess an MMP and probably expresses from the hypothetical genes of other factors that may contribute to the primary infection of SporNPV.

The ratio of SporNPV and SporGV estimated for the wild mixture (98.0%:2.0%) suggests that both viruses naturally coexist in the host in a proportion that allows SporGV to enhance SporNPV without reaching the maximum enhancing potential observed in laboratory conditions (97.5%:2.5%), which could be a strategy to ensure the persistence of both viral agents. The low proportion of SporGV in the wild mixture facilitates the primary infection process for the SporNPV, which rapidly multiplies and dominates the disease development. At the same time, the SporGV multiplies, possibly in a slower rate to avoid driving the process to a granulosis disease, which takes longer and is less transmissible because this GV does not liquefy the host integument. In this way, the SporGV could be improving its transmissibility and persistence in the environment by getting OBs to be released into the environment from liquefied insects [42]. SporGV is a slowly killing betabaculovirus, as demonstrated when used in infections without NPV. This result is a common characteristic of Type I GVs, those that usually only infect the fatty body without causing clear signs of infection or compromising larval growth to their final stages [29]. Classification of SporGV in this category requires further experimental testing. Considering the low mortality of *S. ornithogalli* larvae infected with SporGV, it could be speculated that its permanence in the insect population without another associated virus (SporNPV) is difficult. By contrast, SporNPV probably could persist in nature without coinfection process.

Of the 56 species of NPVs reported in ICTV [43], only in seven cases have GVs been described for the same hosts, out of 26 registered betabaculoviruses. This group includes NPVs (six group 2- and one group 1-alphabaculoviruses) and GVs from *Agrotis segetum* [10,44], *Helicoverpa armigera* [45,46], *Mythimna unipuncta* [47,48], *S. frugiperda* [49,50], *S. litura* [32,51], *Trichoplusia ni* [52,53], and *Choristoneura fumiferana* [54]. All these GVs are of the slowly killing type and a potentiating effect has only been reported to NPVs for *S. frugiperda* [13,55,56]. Probably, coinfection with NPVs is an evolutionary strategy selected in some slowly killing GVs to persist and spread in nature.

## 5. Conclusions

*Spodoptera ornithogalli nucleopolyhedrovirus* (SporNPV) and *Spodoptera ornithogalli granulovirus* (SporGV) must be considered as two novel species of *Baculoviridae* (group II-*Alphabaculovirus* or *Betabaculovirus* genus, respectively) that naturally coinfect *S. ornithogalli* larva in South America, although they could also infect other insects of the *Spodoptera* spp. complex such as *S. frugiperda*. This work shows how the coexistence of evolutionarily distant viruses, but of the same family, can persist in nature, synergizing with each other, and proposes new active ingredients useful for the formulation of bioinsecticides based on baculovirus for the control of the polyphagous crop pest *S. ornithogalli*.

## Figures and Tables

**Table 1:** K2P pairwise distances calculated for betabaculoviruses.

		1	2	3	4	5	6	7	8	9	10	11	12	13	14	15	16	17	18	19	20	21
1	SporGV		0.013	0.011	0.013	0.012	0.012	0.012	0.012	0.012	0.010	0.009	0.010	0.012	0.012	0.010	0.011	0.011	0.009	0.004	0.010	0.010
2	AdorGV	0.969		0.011	0.011	0.009	0.010	0.009	0.011	0.009	0.013	0.012	0.015	0.011	0.010	0.013	0.008	0.013	0.014	0.012	0.013	0.013
3	AgseGV	0.844	0.873		0.012	0.011	0.012	0.010	0.012	0.011	0.011	0.011	0.013	0.012	0.012	0.011	0.010	0.011	0.011	0.011	0.011	0.011
4	ClanGV	0.961	0.812	0.937		0.009	0.008	0.009	0.011	0.009	0.013	0.014	0.013	0.009	0.011	0.014	0.008	0.013	0.013	0.013	0.014	0.013
5	CrleGV	0.903	0.690	0.825	0.738		0.007	0.008	0.011	0.008	0.013	0.012	0.016	0.010	0.009	0.012	0.007	0.013	0.015	0.012	0.012	0.012
6	CypoGV	0.890	0.786	0.875	0.675	0.492		0.009	0.010	0.008	0.012	0.013	0.011	0.008	0.009	0.012	0.007	0.011	0.011	0.013	0.012	0.012
7	DisaGV	0.913	0.734	0.832	0.735	0.620	0.667		0.011	0.008	0.012	0.012	0.015	0.009	0.009	0.012	0.007	0.012	0.013	0.012	0.012	0.012
8	EpapGV	0.921	0.863	0.869	0.863	0.835	0.781	0.833		0.011	0.012	0.012	0.012	0.010	0.011	0.013	0.010	0.011	0.012	0.012	0.013	0.012
9	ErelGV	0.924	0.736	0.858	0.709	0.655	0.648	0.661	0.814		0.013	0.012	0.014	0.010	0.009	0.013	0.007	0.012	0.013	0.012	0.013	0.013
10	HearGV	0.763	0.977	0.865	1.005	0.939	0.927	0.937	0.928	0.954		0.004	0.006	0.012	0.012	0.003	0.011	0.011	0.006	0.009	0.003	0.001
11	MolaGV	0.750	0.937	0.868	1.015	0.902	0.943	0.924	0.934	0.927	0.339		0.006	0.013	0.012	0.005	0.011	0.011	0.006	0.009	0.005	0.004
12	MyunGV	0.755	1.093	0.964	0.970	1.082	0.877	1.045	0.920	1.009	0.488	0.516		0.012	0.014	0.006	0.014	0.011	0.005	0.010	0.006	0.006
13	PlinGV	0.897	0.812	0.876	0.731	0.731	0.663	0.699	0.770	0.724	0.933	0.950	0.876		0.010	0.012	0.009	0.011	0.012	0.012	0.012	0.012
14	PhopGV	0.942	0.745	0.877	0.822	0.693	0.712	0.709	0.831	0.727	0.952	0.942	1.032	0.780		0.013	0.008	0.012	0.013	0.012	0.013	0.013
15	PsunGV	0.764	0.985	0.865	1.008	0.922	0.939	0.926	0.942	0.962	0.223	0.333	0.485	0.936	0.960		0.011	0.011	0.006	0.009	0.000	0.003
16	PiraGV	0.873	0.655	0.795	0.644	0.529	0.576	0.550	0.775	0.536	0.884	0.857	1.035	0.669	0.648	0.880		0.012	0.013	0.011	0.011	0.011
17	PlxyGV	0.871	0.932	0.857	0.950	0.918	0.864	0.919	0.862	0.921	0.873	0.876	0.859	0.870	0.940	0.862	0.864		0.011	0.011	0.011	0.011
18	SpfrGV	0.746	1.036	0.896	0.973	1.012	0.863	0.968	0.921	0.955	0.487	0.512	0.418	0.904	0.997	0.491	0.955	0.855		0.009	0.006	0.006
19	SpltGV	0.309	0.935	0.823	0.991	0.877	0.928	0.891	0.918	0.918	0.739	0.729	0.783	0.928	0.923	0.741	0.848	0.858	0.761		0.009	0.009
20	TrniGV	0.764	0.987	0.865	1.008	0.924	0.940	0.926	0.943	0.962	0.223	0.332	0.484	0.936	0.961	0.003	0.880	0.862	0.491	0.740		0.003
21	XecnGV	0.771	0.983	0.867	1.004	0.937	0.925	0.938	0.933	0.952	0.030	0.339	0.486	0.935	0.958	0.221	0.886	0.876	0.487	0.743	0.221	

Distance values are shown below diagonal. Standard errors are shown above the diagonal. Blue highlighted is the SporGV closest distance value.

**Table 2:** K2P pairwise distances calculated for Group II alphabaculoviruses.

		1	2	3	4	5	6	7	8	9	10	11	12	13	14	15	16	17	18	19	20	21	22	23	24	25	26	27
1	LeseNPV		0.007	0.014	0.011	0.012	0.013	0.013	0.013	0.014	0.014	0.013	0.015	0.013	0.015	0.010	0.012	0.012	0.013	0.012	0.013	0.010	0.010	0.011	0.011	0.012	0.010	0.010
2	SpliNPV	0.635		0.012	0.011	0.013	0.012	0.013	0.012	0.013	0.013	0.013	0.014	0.012	0.013	0.011	0.012	0.012	0.012	0.011	0.011	0.011	0.011	0.011	0.011	0.012	0.011	0.011
3	ClbiNPV	1.067	1.013		0.010	0.010	0.009	0.009	0.009	0.009	0.009	0.009	0.009	0.009	0.010	0.012	0.009	0.009	0.010	0.010	0.010	0.011	0.010	0.010	0.010	0.010	0.010	0.010
4	HearNPV	0.929	0.913	0.839		0.011	0.010	0.010	0.010	0.010	0.010	0.010	0.010	0.010	0.010	0.013	0.010	0.010	0.010	0.010	0.010	0.011	0.011	0.010	0.010	0.010	0.010	0.010
5	LafiNPV	0.983	1.003	0.825	0.895		0.009	0.009	0.008	0.009	0.009	0.009	0.009	0.008	0.011	0.009	0.011	0.011	0.010	0.010	0.010	0.010	0.010	0.010	0.010	0.010	0.011	0.010
6	PeluSNPV	0.995	0.942	0.672	0.818	0.781		0.009	0.009	0.009	0.009	0.009	0.009	0.009	0.010	0.010	0.009	0.009	0.009	0.010	0.010	0.010	0.010	0.009	0.009	0.010	0.009	0.010
7	EcobNPV	1.047	1.007	0.770	0.841	0.776	0.748		0.008	0.008	0.008	0.008	0.008	0.008	0.010	0.012	0.009	0.010	0.010	0.010	0.010	0.011	0.010	0.010	0.010	0.010	0.010	0.010
8	EupsNPV	1.009	0.984	0.756	0.853	0.726	0.734	0.665		0.008	0.008	0.007	0.008	0.007	0.010	0.010	0.010	0.010	0.010	0.010	0.010	0.010	0.010	0.010	0.010	0.010	0.010	0.010
9	BusuNPV	1.058	1.028	0.765	0.837	0.770	0.737	0.649	0.661		0.006	0.008	0.008	0.008	0.010	0.012	0.010	0.010	0.010	0.010	0.010	0.012	0.011	0.010	0.010	0.010	0.010	0.011
10	SujuNPV	1.073	1.049	0.780	0.867	0.808	0.759	0.675	0.693	0.530		0.008	0.008	0.008	0.010	0.012	0.010	0.010	0.010	0.010	0.010	0.011	0.011	0.010	0.010	0.010	0.010	0.011
11	HespNPV	1.022	1.008	0.797	0.861	0.789	0.741	0.665	0.642	0.673	0.685		0.008	0.007	0.010	0.011	0.010	0.010	0.010	0.010	0.010	0.011	0.010	0.010	0.010	0.010	0.010	0.010
12	ApciNPV	1.137	1.055	0.754	0.834	0.801	0.728	0.652	0.674	0.643	0.665	0.665		0.008	0.010	0.014	0.010	0.010	0.010	0.010	0.010	0.013	0.012	0.010	0.010	0.011	0.011	0.012
13	OrleNPV	0.994	0.994	0.760	0.844	0.715	0.732	0.654	0.591	0.632	0.665	0.620	0.657		0.010	0.010	0.010	0.010	0.010	0.010	0.010	0.010	0.010	0.010	0.010	0.010	0.009	0.010
14	AdhoNPV	1.096	1.042	0.836	0.862	0.906	0.808	0.840	0.840	0.830	0.844	0.853	0.804	0.833		0.014	0.010	0.010	0.010	0.010	0.010	0.013	0.012	0.010	0.010	0.010	0.011	0.011
15	LydiMNPV	0.860	0.953	0.896	0.992	0.808	0.829	0.919	0.832	0.915	0.932	0.874	1.043	0.820	1.052		0.012	0.012	0.012	0.012	0.012	0.008	0.008	0.011	0.011	0.011	0.010	0.009
16	TrniSNPV	0.977	0.965	0.793	0.824	0.877	0.767	0.806	0.825	0.804	0.825	0.811	0.794	0.803	0.834	0.942		0.002	0.002	0.008	0.008	0.009	0.008	0.008	0.008	0.008	0.008	0.008
17	ChchNPV	0.967	0.960	0.798	0.827	0.879	0.769	0.807	0.828	0.808	0.845	0.830	0.802	0.815	0.840	0.927	0.164		0.002	0.008	0.008	0.009	0.008	0.008	0.008	0.008	0.008	0.008
18	PsinSNPV	0.977	0.976	0.807	0.826	0.876	0.774	0.817	0.827	0.806	0.848	0.826	0.799	0.806	0.842	0.931	0.176	0.167		0.009	0.008	0.009	0.008	0.009	0.009	0.009	0.008	0.008
19	HearMNPV	0.986	0.972	0.825	0.816	0.857	0.796	0.820	0.818	0.811	0.843	0.843	0.820	0.815	0.863	0.923	0.699	0.695	0.706		0.000	0.007	0.006	0.006	0.006	0.007	0.007	0.006
20	MabrMNPV	0.990	0.976	0.829	0.819	0.861	0.799	0.823	0.822	0.814	0.845	0.844	0.823	0.817	0.863	0.925	0.700	0.697	0.707	0.008		0.007	0.006	0.006	0.007	0.007	0.007	0.006
21	PespNPV	0.819	0.911	0.879	0.907	0.825	0.832	0.893	0.833	0.888	0.919	0.892	0.989	0.827	0.982	0.669	0.749	0.750	0.748	0.549	0.553		0.005	0.006	0.006	0.006	0.006	0.005
22	AgipMNPV	0.844	0.908	0.841	0.857	0.829	0.807	0.861	0.818	0.871	0.892	0.843	0.908	0.806	0.927	0.725	0.705	0.703	0.711	0.527	0.530	0.401		0.005	0.005	0.005	0.005	0.005
23	SfMNPV 3AP2	0.933	0.928	0.830	0.824	0.839	0.794	0.814	0.816	0.822	0.840	0.831	0.830	0.813	0.874	0.883	0.706	0.703	0.717	0.558	0.562	0.552	0.448		0.000	0.001	0.004	0.004
24	SfMNPV Col	0.933	0.928	0.829	0.825	0.839	0.794	0.814	0.816	0.821	0.839	0.829	0.829	0.814	0.873	0.883	0.707	0.703	0.718	0.558	0.562	0.552	0.447	0.005		0.001	0.004	0.004
25	SporNPV	0.931	0.933	0.831	0.830	0.841	0.801	0.819	0.815	0.827	0.840	0.831	0.837	0.813	0.877	0.873	0.712	0.705	0.718	0.560	0.564	0.548	0.441	0.025	0.021		0.004	0.004
26	SpexMNPV	0.906	0.902	0.838	0.835	0.859	0.798	0.825	0.816	0.837	0.865	0.831	0.864	0.807	0.890	0.810	0.699	0.704	0.705	0.560	0.556	0.503	0.407	0.360	0.360	0.343		0.003
27	SpltMNPV	0.869	0.902	0.831	0.842	0.838	0.806	0.832	0.812	0.852	0.880	0.829	0.894	0.811	0.901	0.767	0.704	0.705	0.706	0.559	0.562	0.472	0.384	0.343	0.342	0.323	0.205	

Distance values are shown below diagonal. Standard errors are shown above the diagonal. Blue highlighted is the SporMNPV closest distance values.

**Table 3:** Mean lethal concentrations (LC_50_) and confidence intervals (95%) of the wild viral mixture in neonate larvae of *S. ornithogalli* and *S. frugiperda* at 7 days after inoculation.

Insect	Slope (±SE)	LC_50_ (OBs/mL)	Fiducial Limits (95%)		df	χ^2^
			Low	High		
*S. ornithogalli*	1.29 (0.15)	4.87 × 10^4^	2.88 × 10^4^	8.24 × 10^4^	3	5.22

**Table 4:** Mean lethal concentrations (LC_50_), confidence intervals (95%), and relative potency of SporMNPV and its artificial mixtures with SporGV in neonate *S. ornithogalli* larvae. M1: SporMNPV 90% and SporGV 10%, M2: SporMNPV 95% and SporGV 5%, M3: SporMNPV 97.5% and SporGV 2.5%. Mortality data for analysis were recorded 7 days after inoculation. LC_50_ values designated by different letters are significantly different due to non-overlap of 95% fiducial limits. The *p*-values lower than 0.05 indicate significant differences in comparison with SporNPV.

**Treatment**	**Slope (±SE)**	**LC_50_ (OBs/mL)**	**Relative Potency**	**Fiducial Limits** **(95%)**		***p* Value**
				**Low**	**High**	
SporNPV	1.03 (0.11)	1.66 × 10^5^ a		1.04 × 10^5^	2.60 × 10^5^	
M1	1.04 (0.12)	1.23 × 10^5^ ab	1.35	7.17 × 10^4^	2.11 × 10^5^	0.05
M2	1.38 (0.16)	7.39 × 10^4^ ab	2.25	4.22 × 10^4^	1.28 × 10^5^	0.45
M3	1.38 (0.18)	5.42 × 10^4^ b	3.06	3.03 × 10^4^	9.48 × 10^4^	<0.01

## Data Availability

The data presented in this study are available in Supplementary Table S1.

## Supplementary Materials

The following are available online at https://www.mdpi.com/article/10.3390/v13122520/s1. Table S1: Sequences used in bioinformatics’ analysis.
