# Supplementary material for: Natural Coinfection between Novel Species of Baculoviruses in Spodoptera ornithogalli Larvae
Source: Viruses. 2021 Dec 15;13(12):2520. doi: 10.3390/v13122520 (PMC8703766; doi:10.3390/v13122520)
Supplement: Supplementary file 1 [file viruses-13-02520-s001.zip › viruses-1489846-supplementary.pdf]

Supplementary Materials

Table S1. Sequences used in bioinformatics analysis.

Genus/ Group	Nomenclature	Abbreviation	Isolate	Accesion no.
Alpha Group I	*Antheraea pernyi nucleopolyhedrovirus*	AnpeNPV	Z	NC_008035
	Anticarsia gemmatalis multiple nucleopolyhedrovirus	AgMNPV	2D	NC_008520
	Autographa californica multiple nucleopolyhedrovirus	AcMNPV	Clone C6	NC_001623
	Bombyx mori nucleopolyhedrovirus	BmNPV	T3	NC_001962
	Catopsilia pomona	CapoNPV	416	NC_030240
	Choristoneura fumiferana multiple nucleopolyhedrovirus	CfMNPV		NC_004778
	Condylorrhiza vestigialis multiple nucleopolyhedrovirus	CoveMNPV		NC_026430
	Dasychira pudibunda nucleopolyhedrovirus	DapuNPV	ML1	KP747440
	Dendrolimus kikuchii nucleopolyhedrovirus	DekiNPV	strain YN	JX193905
	Epiphyas postvittana nucleopolyhedrovirus	EppoNPV		NC_003083
	Hyphantria cunea nucleopolyhedrovirus	HycuNPV		NC_007767
	Lonomia obliqua multiple nucleopolyhedrovirus	LoobMNPV	SP/2000	KP763670
	Maruca vitrata nucleopolyhedrovirus	MaviNPV		NC_008725
	Orgyia pseudotsugata multiple nucleopolyhedrovirus	OpMNPV		NC_001875
	Oxyplax ochracea nucleopolyhedrovirus	OxocNPV	435	MF143631
	Philosamia cynthia ricini nucleopolyhedrovirus	PhcyNPV		JX404026
	Plutella xylostella multiple nucleopolyhedrovirus	PlxyMNPV	CL3	NC_008349
	Rachiplusia ou multiple nucleopolyhedrovirus	RoMNPV		NC_004323
	Samia cynthia nucleopolyhedrovirus	SacyNPV	Nagano	LC375538
	Spilosoma obliqua nucleopolyhedrovirus	SpobNPV	IIPR	KY550224
	Thysanoplusia orichalcea nucleopolyhedrovirus	ThorNPV	P2	NC_019945
	Adoxophyes honmai nucleopolyhedrovirus	AdhoNPV		NC_004690

Alpha Group II	*Agrotis ipsilon multiple nucleopolyhedrovirus*	AgipMNPV		NC_011345
	Apocheima cinerarium nucleopolyhedrovirus	ApciNPV		NC_018504
	Buzura suppressaria nucleopolyhedrovirus	BusuNPV	Hubei	NC_023442
	Chrysodeixis chalcites nucleopolyhedrovirus	ChchNPV		NC_007151
	Clanis bilineata nucleopolyhedrovirus	ClbiNPV	DZ1	NC_008293
	Ectropis obliqua nucleopolyhedrovirus	EcobNPV	A1	NC_008586
	Euproctis pseudoconspersa nucleopolyhedrovirus	EupsNPV		NC_012639
	Helicoverpa armigera multiple nucleopolyhedrovirus	HaMNPV		NC_011615
	Helicoverpa armigera nucleopolyhedrovirus	HaSNPV-G4	G4	NC_002654
	Hemileuca sp nucleopolyhedrovirus	HespNPV		NC_021923
	Lambdina fiscellaria nucleopolyhedrovirus	LafiNPV-GR15	GR15	NC_026922
	Leucania separata nucleopolyhedrovirus	LeseNPV	AH1	NC_008348
	Lymantria dispar multiple nucleopolyhedrovirus	LdMNPV		NC_001973
	Mamestra brassicae multiple nucleopolyhedrovirus	MabrMNPV-K1	K1	NC_023681
	Orgyia leucostigma nucleopolyhedrovirus	OrleNPV	CFS77	NC_010276
	Peridroma sp nucleopolyhedrovirus	PespNPV	GR-167	NC_024625
	Perigonia lusca simple nucleopolyhedrovirus	PeluSNPV		NC_027923
	Pseudoplusia includens simple nucleopolyhedrovirus	PsinSNPV-IE	IE	NC_026268
	Spodoptera exigua multiple nucleopolyhedrovirus	SeMNPV		NC_002169
	Spodoptera frugiperda multiple nucleopolyhedrovirus	SfMNPV-3AP2	3AP2	NC_009011
	Spodoptera littoralis nucleopolyhedrovirus	SpliNPV-AN1956	AN1956	JX454574
	Spodoptera litura II multiple nucleopolyhedrovirus	SpltMNPV-II		NC_011616
	Sucra jujuba nucleopolyhedrovirus	SujuNPV-473	473	KJ676450
	Trichoplusia ni simple nucleopolyhedrovirus	TnSNPV		NC_007383
	Spodoptera frugiperda multiple nucleopolyhedrovirus	SfMNPV-Col	Colombia	KF891883
Beta	*Adoxophyes orana granulovirus*	AdorGV		NC_005038
	Agrotis segetum granulovirus	AgseGV		NC_005839
	Clostera anachoreta granulovirus	ClanGV	HBHN	NC_015398

	Cryptophlebia leucotreta granulovirus	CrleGV		NC_005068
	Cydia pomonella granulovirus	CpGV		NC_002816
	Diatraea saccharalis granulovirus	DisaGV	Parana-2009	NC_028491
	Epinotia aporema granulovirus	EpapGV		NC_018875
	Erinnyis ello granulovirus	ErelGV	BrS86	NC_025257
	Helicoverpa armigera granulovirus	HearGV		NC_010240
	Mocis latipes granulovirus	MolaGV	Southern Brazil	NC_029996
	Mythimna unipuncta granulovirus	MyunGV	#8	NC_033780
	Phthorimaea operculella granulovirus	PhopGV		NC_004062
	Pseudaletia unipuncta granulovirus	PsunGV	Hawaiin	NC_013772
	Pieris rapae granulovirus	PiraGV	Wuhan	NC_013797
	Plodia interpunctella granulovirus	PlinGV	Cambridge	NC_032255
	Plutella xylostella granulovirus	PxGV		NC_002593
	Spodoptera frugiperda granulovirus	SpfrGV	VG008	NC_026511
	Spodoptera litura granulovirus	SpltGV	K1	NC_009503
	Trichplusia ni granulovirus	TrniGV	LBIV-12	KU752557
	Xestia c nigrum granulovirus	XecnGV		NC_002331
Gamma	*Neodiprion lecontei nucleopolyhedrovirus*	NeleNPV		NC_005906
	Neodiprion sertifer nucleopolyhedrovirus	NeseNPV		NC_005905
	Neodiprion abietis nucleopolyhedrovirus	NeabNPV		DQ317692
Delta	*Culex nigripalpus nucleopolyhedrovirus*	CuniNPV	Florida 1997	NC_003084

Alpha: *Alphabaculovirus*; Beta: *Betabaculovirus*; Gamma: *Gammabaculovirus*; Delta: *Deltabaculovirus*.
